# Supplementary material for: Acute Effects of the Consumption of Passiflora setacea Juice on Metabolic Risk Factors and Gene Expression Profile in Humans
Source: Nutrients. 2020 Apr 16;12(4):1104. doi: 10.3390/nu12041104 (PMC7231153; doi:10.3390/nu12041104)
Supplement: Supplementary file 1 [file nutrients-12-01104-s001.pdf]

List S1: Ingredients, in descending order, of the isotonic drink: water, sucrose, maltodextrin, sodium chloride, sodium citrate, potassium phosphate monobasic, citric acid acidulant, tartrazine artificial flavoring and FD&C Yellow 6 (dye).

Table S1: *White meal* composition:

Meal	Menu
Breakfast	French bread, cheese, ham and milk.
Snack	Cake made from flour, eggs, milk, sugar and yeast.
Lunch	Rice, beans, toasted cassava flour, meat and soda.
Snack	French bread, cheese, ham and milk or soda.
Dinner	Rice, beans, toasted cassava flour and meat.

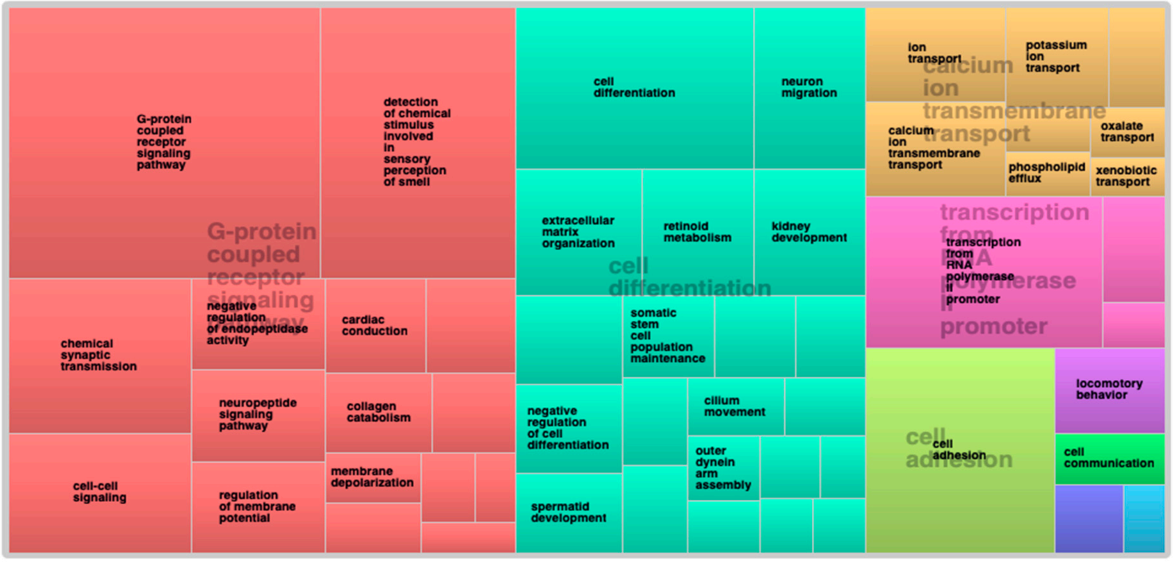

Figure S1. Gene Ontology terms grouped by REVIGO. The mRNA of PBMC's subjects was submitted to microarray analyses after their intake of the *Passiflora setacea* juice and the placebo drink. Differentially expressed genes based on gene ontology (GO) and grouped by REVIGO revealed biological processes (calcium ion transmembrane transport, cell differentiation, G-protein coupled receptor signaling pathway, cell adhesion and transcription from RNA polymerase promoter) clusters of down-regulated genes.

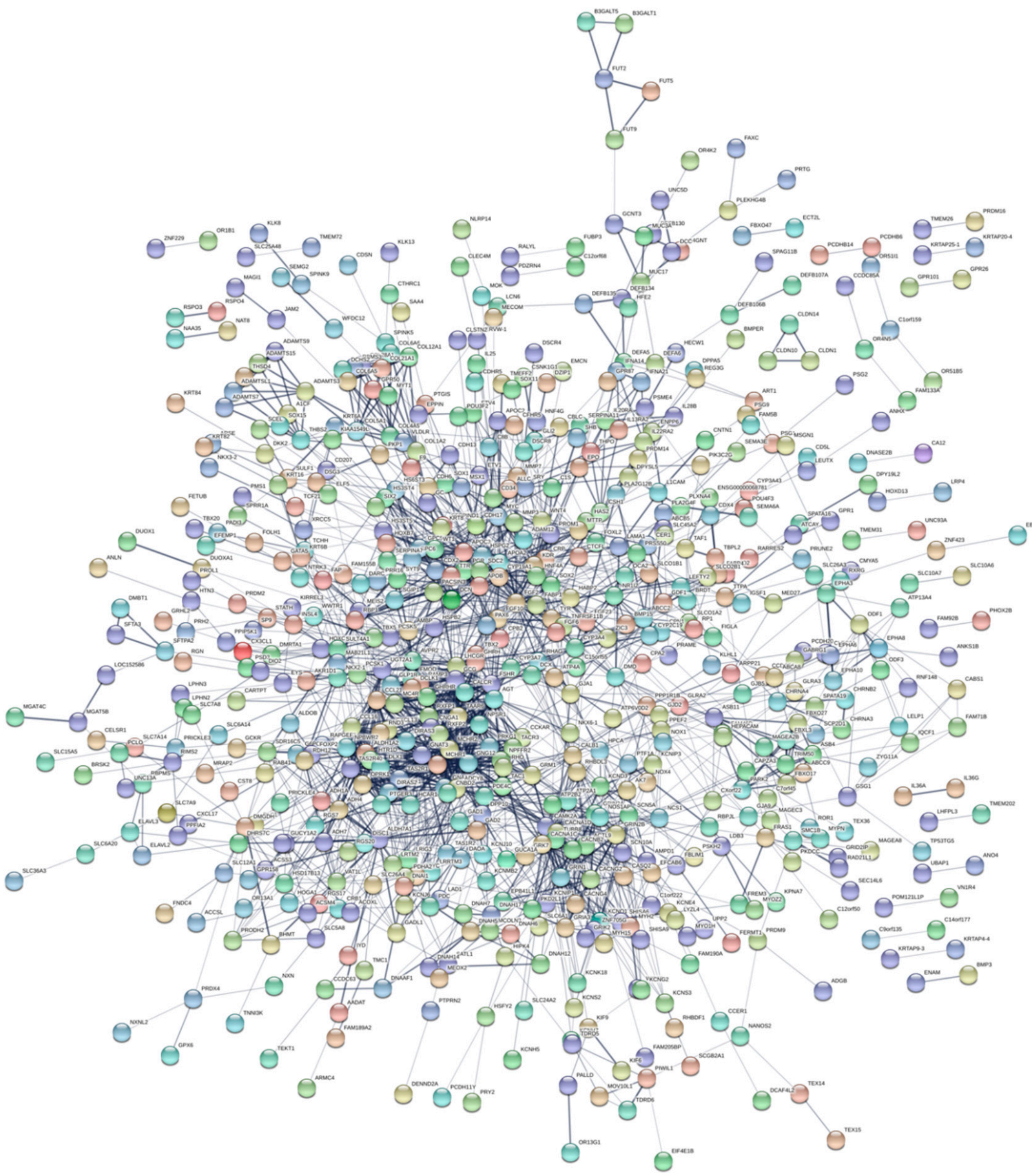

Figure S2. Bioinformatics analyses of protein-protein interactions of the genes whose expression have been affected by the consumption of *Passiflora setacea* (Online String database).
